# Contemporary Trends in Malignant Peritoneal Mesothelioma: Incidence and Survival in the United States

**DOI:** 10.3390/cancers15010229

**Published:** 2022-12-30

**Authors:** Lucia Calthorpe, Fernanda Romero-Hernandez, Phoebe Miller, Patricia C. Conroy, Kenzo Hirose, Alex Kim, Kimberly Kirkwood, Eric Nakakura, Carlos Corvera, Ajay V. Maker, Adnan Alseidi, Mohamed Abdelgadir Adam

**Affiliations:** 1 Department of Surgery, University of California, San Francisco, CA 94143, USA; 2 Department of Surgery, Division of Surgical Oncology, University of California, San Francisco, CA 94143, USA; 3 Department of Surgery, Division of Surgical Oncology, The Ohio State University, Columbus, OH 43210, USA

**Keywords:** mesothelioma, cytoreductive surgery, hyperthermic intraperitoneal chemotherapy

## Abstract

**Simple Summary:**

This contemporary epidemiological analysis revealed a stable overall incidence of malignant peritoneal mesothelioma (MPM), but improved recognition of epithelioid histology. Survival improved over time and was associated with cancer-directed surgery, suggesting that accurate diagnosis of epithelioid histology may lead to more patients being considered for appropriate multimodal treatment and contribute to improved overall survival.

**Abstract:**

Background: Malignant peritoneal mesothelioma (MPM) is a rare disease with a historically poor prognosis. Given the emergence of effective therapies, a contemporary analysis of MPM incidence and survival is warranted. Methods: The SEER-18 registry dataset was analyzed (2000–2018). Age-adjusted annual incidence was stratified by sex and histology. Joinpoint regression was used to estimate annual percent change (APC) in incidence. Multivariable cox proportional hazards models were used to investigate survival trends. Results: Of 1689 MPM cases, most were male (55.4%), >50 years (80.0%), and white (75.2%). Age-adjusted incidence of MPM remained stable over time, with an average annual incidence of 1.02 cases/million. Epithelioid histology increased by 240% (APC 2.6; 95% CI: 0.7, 4.5), while incidence of undefined histology decreased significantly (APC −2.1; 95% CI: −3.1, −1.1). Cases treated with cancer-directed surgery increased from 27% to 43%. Overall median age-standardized survival was 11.6 months. Median age-standardized survival was 16.6 months for epithelioid histology but 2.0 months for sarcomatoid histology. Diagnosis in recent years (2015–2018 HR 0.51; 95% CI: 0.38, 0.67) and receipt of cancer-directed surgery (HR 0.84; 95% CI: 0.72, 0.98) were associated with improved survival. Conclusions: Although the overall incidence of MPM remained stable, recognition of epithelioid histology increased. Concurrent with an increase in cancer-directed surgery, MPM survival has improved.

## 1. Introduction

Malignant peritoneal mesothelioma (MPM) is a rare clinical entity accounting for 10–30% of mesothelioma cases diagnosed in the United States [1]. Mesothelioma is derived from mesothelial cells lining serous cavities, is more common in males, and is associated with asbestos exposure [2]. However, recent studies suggest that the association between peritoneal mesothelioma and asbestos is less strong than the well-established link between asbestos and pleural mesothelioma [3,4,5,6,7].

MPM prognosis is generally poor, with a median survival of less than one year [8,9]. Poor prognostic factors include high Ki-67 levels, thrombocytosis, lymph node metastases at diagnosis, and histologic differentiation other than epithelioid [10]. Epithelioid is the most common histology and is generally associated with superior survival [11]. Sarcomatoid histology is less common and is associated with more aggressive behavior and poor response to treatment [12]. Biphasic, or mixed type, has both epithelioid and sarcomatoid features and is difficult to treat with overall poor prognosis [3]. 

Historically, the incidence of MPM has been reported to be 0.1 per 100,000 person-years [13]. However, the most recent studies examining the incidence of MPM were conducted in the early 2000s and did not consider trends in incidence over time or differences by sex and histology [13,14]. In recent years, cytoreductive surgery (CRS) and hyperthermic intraperitoneal chemotherapy (HIPEC) have emerged as viable therapies for MPM and have improved our ability to treat appropriately selected patients [12]. Although first described in the late 1980’s, a significant increase in the use of CRS-HIPEC to treat peritoneal malignancies has been observed over the last decade [14]. A recent study examining survival of patients with pleural or peritoneal mesothelioma showed that CRS/HIPEC procedures extended the overall survival from a median of 6 months to 34–92 months in treatment-naïve patients [15]. 

Given the emergence of effective therapies and the limitations of existing literature, a contemporary analysis of trends in MPM incidence and survival is warranted. The aims of this study were to (1) describe the trends in the incidence of MPM from 2000 to 2018 in the United States, stratifying by sex and histology; and (2) describe national trends in survival, controlling for histologic type, age, sex, race, stage, and receipt of cancer-directed surgery.

## 2. Materials and Methods

### 2.1. Data Source

MPM cases were identified from the Surveillance, Epidemiology and End Results Program (SEER) 18 registry dataset from 2000 to 2018 [16]. SEER is a program of the National Cancer Institute and represents the largest data source on cancer incidence and survival in the United States. SEER-18 covers approximately 27.8% of the United States population (based on the 2010 census) and contains information on 8,666,662 tumors [17]. The following geographic regions are included in SEER-18: Alaska, Atlanta, California, Connecticut, Detroit, Georgia, Hawaii, Iowa, Kentucky, Louisiana, New Mexico, New Jersey, Seattle, and Utah [17].

### 2.2. Study Population-Malignant Peritoneal Mesothelioma

MPM cases were identified based on the International Classification of Diseases for Oncology, Third Edition (ICD-O-3) [18]. Cases were included if histology was reported as sarcomatoid (9051/3), biphasic (9053/3), epithelioid (9052/3), or malignant mesothelioma, not otherwise specified (9050/3). Patients were considered to have peritoneal mesothelioma if their site of malignancy was coded as peritoneum/retroperitoneum (48.0–48.8); connective tissue of the abdomen (49.4); ovary (56.9); or abdomen, not otherwise specified (76.2). Patients were excluded from survival analysis if MPM was not their first primary cancer diagnosis, or survival time was unknown. This study was granted exemption by our Institutional Review Board given the de-identified nature of the data.

### 2.3. Clinical and Demographic Variables 

The following demographic variables were extracted: age, sex, race/ethnicity (white, Black, Hispanic, other), and year of diagnosis (2000–2018). Clinical variables extracted included histology (epithelioid, biphasic, sarcomatoid, and undefined), anatomic site (peritoneum and retroperitoneum, soft tissue of the abdomen, and ovary), stage (localized, regional, distant, and unknown), and receipt of cancer-directed surgery. Consistent with prior literature, cancer-directed surgery was defined as (1) partial or total removal of primary site, (2) debulking surgery, or (3) radical surgery (primary site removal with resection in continuity with other organs) [19]. 

### 2.4. Statistical Analysis

Descriptive statistics were tabulated and expressed as counts and percentages. Overall unadjusted annual incidence of MPM was computed. Joinpoint regression using the weighted least squares method was used to compute the annual percent change (APC) in MPM incidence and assess for changes in trends over time. This analysis was repeated for overall age-adjusted annual incidence (direct method of adjustment to the 2000 United States standard population) and age-adjusted incidence stratified by sex and histology. A multivariable Cox proportional hazards model was used to examine the association between year of diagnosis and overall survival, adjusting for histology, age, sex, race, stage, and receipt of cancer-directed surgery. 

Incidence estimates were computed using SEER*Stat [20]. Joinpoint regression analysis was performed using Joinpoint Trend Analysis Software version 4.9.0.0 [21]. Data was exported from SEER*Stat to STATA/IC version 16.1 for survival analysis [22]. Statistical significance was set at *p* < 0.05 and all tests were two-sided.

## 3. Results

From 2000 to 2018, 1689 new cases of MPM were identified. The majority of cases were in men (55.4%). The most commonly reported race was white (75.2%), and most patients were aged 50 years or older at the time of diagnosis (80.2%). Epithelioid was the most frequently identified histology (37.4%); however, histology was undefined for the majority of patients (55.7%). Cancer-directed surgery was performed in a minority of patients (32.7%) (Table 1).

### 3.1. Incidence Trends

**Figure 1:**
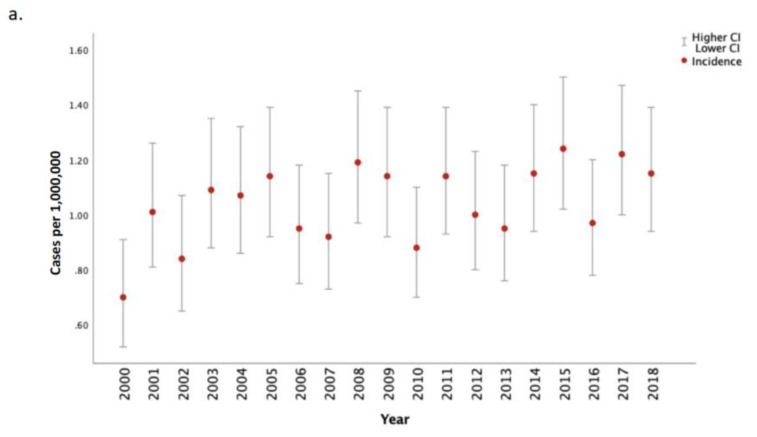

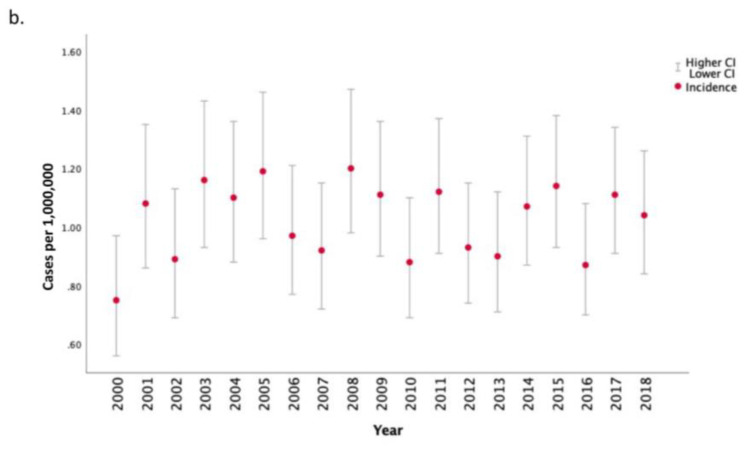
Overall Unadjusted (part (**a**)) and Age-Adjusted (part (**b**)) Incidence of MPM from 2000 to 2018. Red dots represent annual incidence estimates (per 1,000,000 person-years), grey bars represent 95% confidence intervals. Rates are per 1,000,000 and age-adjusted to the 2000 US Std Population (19 age groups—Census P25-1130) standard.

The average age-adjusted incidence rate of MPM between 2000–2018 was 1.02 cases per 1,000,000 person-years. The unadjusted annual incidence of MPM increased over the study period (Percent change [PC] 64.9; APC 1.2; 95% CI: 0.07, 2.31, *p* = 0.038), while the annual age-adjusted incidence of MPM remained stable over time (PC 38.9; APC 0.1; 95% CI −1.0, 1.3, *p* = 0.85) (Figure 1, Table 2).

**Figure 2:**
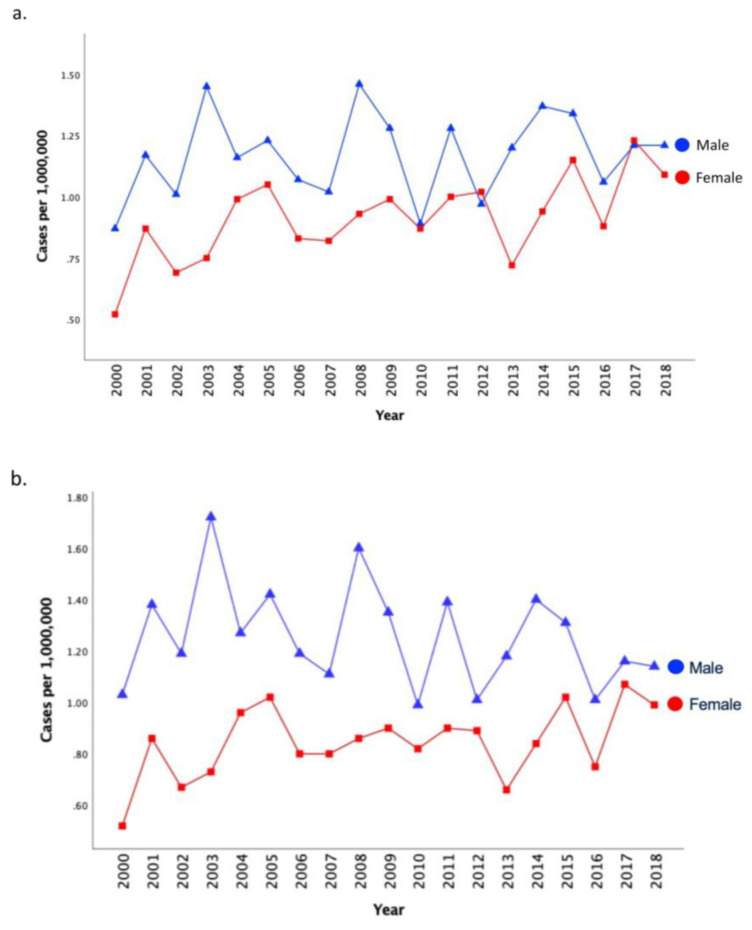
Unadjusted (part (**a**)) and Age-Adjusted (part (**b**)) Incidence of MPM by Sex from 2000 to 2018**.** Blue line represents annual incidence estimates (per 1,000,000 person years) among males, red line represents annual incidence estimates (per 1,000,000 person years) among females. Rates are per 1,000,000 and age-adjusted to the 2000 US Std Population (19 age groups—Census P25-1130) standard.

Unadjusted incidence of MPM increased over time for females (PC 107.9; APC 2.1; 95% CI: 0.7, 3.4, *p* = 0.005), but remained stable for males (PC 38.4; APC 0.5; 95% CI: −0.8, 1.8, *p* = 0.451). Overall age-adjusted incidence among females was 0.85 per 1,000,000 and 1.24 per 1,000,000 among males. There were no statistically significant trends in incidence by sex with age adjustment (Figure 2, Table 2). 

**Figure 3:**
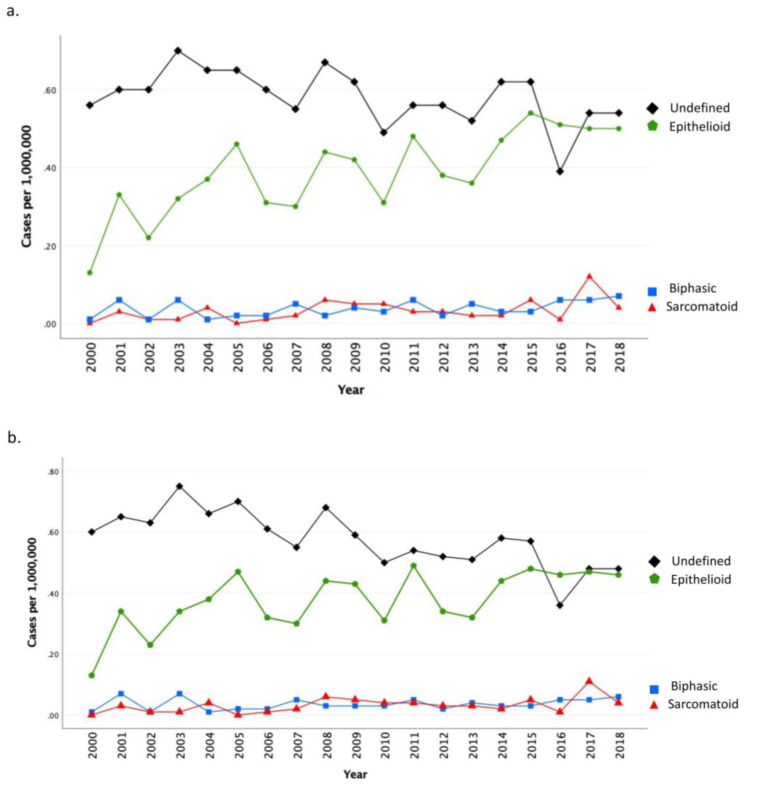
Unadjusted (part (**a**)) and Age-Adjusted (part (**b**)) Annual Incidence of MPM by Histology from 2000 to 2018. Black line represents annual incidence estimates (per 1,000,000 person years) among undefined histology, green line represents annual incidence estimates (per 1,000,000 person years) among epithelioid histology, blue line represents annual incidence estimates (per 1,000,000 person years) among biphasic histology, red line represents annual incidence estimates (per 1,000,000 person years) among sarcomatoid histology. Rates are per 1,000,000 and age-adjusted to the 2000 US Std Population (19 age groups—Census P25-1130) standard.

There was a statistically significant increase in cases with epithelioid histology over time (age-adjusted PC 239.7; APC 2.6; 95% CI: 0.7, 4.5, *p* = 0.01), concurrent with a decrease in cases with undefined histology (age-adjusted PC −19.0; APC −2.1; 95% CI: −3.1, −1.1, *p* = 0.001; Figure 3; Table 2).

### 3.2. Survival Trends

After excluding patients who had missing survival data (*n* = 12) or who’s first primary cancer was not MPM (*n* = 383), 1294 individuals were included in the survival analysis. Of these, the median age-standardized survival was 11.6 months, and 951 (73.5%) individuals died during the study period. Although the median age-standardized survival was 16.6 months for patients with epithelioid histology, it was 2.0 months for those with sarcomatoid histology. From 2000 to 2018, cases treated with cancer-directed surgery increased from 27% to 43%.

Multivariable cox proportional hazards models revealed a statistically significant association between the year of diagnosis and survival, with those diagnosed from 2015 to 2018 having a hazard ratio of 0.51 (95% CI: 0.38, 0.67, *p* < 0.001) compared to those diagnosed from 2000 to 2002 (Table 3). Female sex was associated with a significant decrease in risk of death (HR 0.66; 95% CI: 0.58, 0.76, *p* < 0.001), whereas increasing age was associated with increased risk of death (HR 1.55; 95% CI: 1.44, 1.67, *p* < 0.001). Relative to epithelioid histology, both sarcomatoid and biphasic histology were associated with an increased risk of death (biphasic HR 1.49; 95% CI: 1.03, 2.13, *p* = 0.03; sarcomatoid HR 2.85; 95% CI: 1.99, 4.08, *p* < 0.001). Receipt of cancer-directed surgery was associated with improved survival (HR 0.84; 95% CI: 0.72, 0.98, *p* = 0.02). 

## 4. Discussion

In this large contemporary analysis, specific to peritoneal mesothelioma, the unadjusted incidence of MPM increased from 2000 to 2018. This increase was driven primarily by new diagnoses in women. However, after adjustment for age, the incidence of MPM remained stable throughout the study period. There was an increase in the diagnosis of epithelioid histology with a concurrent decrease in the incidence of unspecified histology. Even after adjusting for factors known to be key determinants of prognosis, including histology, diagnosis in later years (2015–2018) was strongly associated with improved overall survival. 

Despite evidence that MPM is a distinct clinical entity from pleural mesothelioma, several foundational studies examined the incidence of both pleural and peritoneal mesothelioma together [1,23,24,25]. Using the 1999–2002 SEER registry, Larson et al. reported 1.1 mesothelioma cases (including both pleural and peritoneal) per 100,000 persons [25]. Henley et al. reported a similar average of 1.05 mesothelioma (pleural and peritoneal) cases per 100,000 persons diagnosed using the 2003–2008 SEER registry. Between 2003–2008, the age-adjusted incidence of mesothelioma (pleural and peritoneal) decreased by 2.6% per year for men but was stable among women [13]. In contrast, an analysis of the 1973–2005 SEER demonstrated no temporal trends in the age-adjusted incidence of MPM but a decrease in the age-adjusted incidence of pleural mesothelioma [2]. These discordant results are consistent with the notion that MPM and pleural mesothelioma are epidemiologically distinct and should be considered separately. The present study provides an updated analysis of the distinct incidence of MPM, reflecting nearly fifteen additional years of data. 

Similar to the available literature on MPM incidence, existing analyses examining MPM survival are outdated, particularly in light of the increased adoption of effective therapies for MPM over the past decade. Although morbidity after CRS/HIPEC remains high at approximately 55%, studies have consistently shown a decrease in mortality for peritoneal surface malignancies after CRS/HIPEC [10,14,15,26,27]. CRS/HIPEC is now considered standard-of-care for patients with epithelioid mesothelioma (or extremely well-selected biphasic or sarcomatoid mesothelioma) who are appropriate surgical candidates [10].

The most recent MPM survival analysis in SEER includes data through 2011, where Shavelle et al. identified 1634 cases of MPM [3,28]. They found improved survival was associated with female sex, younger age at diagnosis, and surgical intervention, which is similar to the results of the present study. When analyzed as a continuous variable, year of diagnosis had a minimal effect on survival for patients diagnosed with MPM between 1973–2011 (HR 0.98; *p* < 0.001) [3]. In contrast, we found that diagnosis from 2015 to 2018 was associated with improved survival compared to diagnosis from 2000 to 2002 (HR 0.51; *p* < 0.001), suggesting that survival from MPM may have increased in recent years. It is possible that increased adoption of CRS/HIPEC has contributed to this increase in survival. Further, it is possible that CRS/HIPEC itself has become more effective as a result of optimization of chemotherapy agents, and safer as a procedure over time [29].

Ullah et al. also recently reported an analysis of trends in malignant peritoneal mesothelioma based on SEER data [28]. However, despite inclusion of the word incidence in the title of their paper, Ullah et al. do not provide any estimates of the incidence of MPM. Furthermore, they do not analyze trends in incidence by key factors such as sex and histology. Second, the authors describe their analysis as a survival analysis but provide only the odds ratios associated with four variables without specifying covariates or model type. Our study adds depth to the literature with a robust analysis of trends in incidence and a contemporary exploration of factors associated with survival.

We observed an increase in the diagnosis of epithelioid MPM histology during the study period and a concurrent decrease in the incidence of undefined MPM histology. This trend may suggest improvement in the pathological diagnosis of MPM over time. Especially given the overall stable incidence of MPM, it is likely that these concurrent trends reflect more accurate recognition of epithelioid histology. This may be due to factors such as the development and dissemination of consensus guidelines on the diagnosis and treatment of MPM, and recent advances in immunohistochemical marker panels, including BAP1, which have improved pathologists’ ability to differentiate MPM from other tumors with similar features [30,31,32,33]. However, the substantial proportion of remaining cases with undefined histology may suggest a persistent lack of widespread knowledge of the appropriate work up of this rare malignancy.

Limitations of this study include its retrospective and observational nature. Data for SEER is collected at the time of diagnosis and initial treatments. As such, remission and relapse information are not currently included in SEER data collection. Further, SEER does not record variables such as peritoneal cancer index (PCI). SEER does not include data regarding the use of systemic chemotherapy; therefore, conclusions regarding the impact of treatments like HIPEC cannot be drawn definitively. Additionally, cancer-directed surgery included patients who underwent both partial resection and complete resection, potentially limiting our ability to detect the full effect of complete cytoreduction [34]. Finally, it is possible that errors in coding exist in the dataset. Despite these limitations, SEER represents the most comprehensive US-based source of cancer-specific outcomes and is a rich source of epidemiological data on incidence and survival patterns for this rare malignancy. 

The observed increase in epithelioid histology and concurrent decrease in undefined histology has important implications. Accurate diagnosis of MPM is essential to facilitate expedient referral and appropriate initiation of multimodal treatment for MPM. Given the more favorable prognosis of epithelioid histology, CRS/HIPEC is recommended for patients with epithelioid MPMs and only for very select patients with biphasic or sarcomatoid MPM [10]. Consequently, more accurate diagnosis of epithelioid histology may lead to more patients being considered for appropriate multimodal treatment and could contribute to improved overall survival. Our finding of an increase in cancer-directed surgery is consistent with this potential mechanism of improved survival. Future studies examining practice patterns in the use of CRS/HIPEC in MPM are warranted.

## 5. Conclusions

Although the overall age-adjusted incidence of MPM remained stable from 2000 to 2018, there has been an increase in cancer-directed surgery and a concurrent improvement in MPM survival over time. This contemporary analysis of MPM survival reflects trends after increased adoption of effective therapies for MPM, including CRS/HIPEC. Future studies are needed to examine how changes in management patterns, including surgery and systemic therapy, have influenced MPM survival over time. 

## Figures and Tables

**Table 1:** Demographic Characteristics of Patients with Malignant Peritoneal Mesothelioma (MPM) in SEER (2000–2018).

	MPM
	(*n* = 1689)
**Sex**	
Male	937 (55.4%)
Female	752 (44.5%)
**Age**	
0–19	5 (0.3%)
20–34	71 (4.2%)
35–49	259 (15.3%)
50–64	530 (31.3%)
65–79	621 (36.7%)
>80	203 (12.0%)
**Race/Ethnicity**	
White	1271 (75.2%)
Black	99 (5.8%)
Hispanic	228 (13.5%)
Other	86 (5.0%)
**Year of Diagnosis**	
2000–2002	204 (12.1%)
2003–2005	271 (16.0%)
2006–2008	257 (15.2%)
2009–2011	272 (16.1%)
2012–2014	273 (16.2%)
2015–2018	412 (24.4%)
**Histology**	
Epithelioid	632 (37.4%)
Biphasic	62 (3.6%)
Sarcomatoid	54 (3.2%)
Undefined	941 (55.7%)
**Site**	
Peritoneum and Retroperitoneum	1641 (97.2%)
Abdomen and Soft tissue	28 (1.6%)
Ovary	20 (1.1%)
**Stage**	
Localized	200 (11.8%)
Regional	232 (13.7%)
Distant	741 (43.8%)
Unknown	516 (30.5%)
**Extent of Resection**	
Removal of Primary Site	137 (8.0%)
Debulking	270 (15.9%)
Radical	145 (8.5%)

**Table 2:** Unadjusted and adjusted total percent change (PC) and annual percent change (APC) by sex and histology of patients with MPM identified in SEER-18 registries from 2000 to 2018.

	PC	APC (95% CI)	*p* Value
**Unadjusted Overall**	64.9	1.2 * (0.07, 2.31)	0.038
**Adjusted Overall**	38.9	0.1 (−1.0, 1.3)	0.850
**Unadjusted Sex**			
Male	38.4	0.5 (−0.8, 1.8)	0.451
Female	107.9	2.1 * (0.7, 3.4)	0.005
**Adjusted Sex**			
Male	10.5	−0.9 (−2.2, 0.5)	0.200
Female	88.6	1.3 (−0.0, 2.7)	0.060
**Unadjusted Histology**			
Epithelioid	292.5	3.7 * (1.9, 5.5)	<0.001
Biphasic	423.3	2.2 (−1.6, 6.2)	0.238
Undefined	−2.9	−1.0 * (−2.0, −0.02)	0.046
Sarcomatoid	-	-	
**Adjusted Histology**			
Epithelioid	239.7	2.6 * (0.7, 4.5)	0.010
Biphasic	325.1	1.1 (−2.8, 5.1)	0.560
Undefined	−19	−2.1 * (−3.1, −1.1)	0.001
Sarcomatoid	-	-	

APCs were calculated using weighted least squares method. Omitted fields mean statistic could not be calculated based on low *n*. * The APC is significantly different from zero (*p* < 0.05). PC: percent change; APC: annual percent change; CI: confidence interval.

**Table 3:** Multivariable Cox Proportional Hazards model of survival trends of patients with MPM (*n* = 1294).

	HR	*p*-Value	95% CI
**Sex**			
Male (reference)	-		
Female	0.66	<0.001	(0.58, 0.76)
**Age ^a^**	1.55	<0.001	(1.44, 1.67)
**Race/Ethnicity**			
Other (reference) White Black Hispanic (all races)	- 0.97 0.86 1.11	0.821 0.472 0.539	(0.72, 1.30) (0.57, 1.30) (0.80, 1.55)
**Year of diagnosis**			
2000–2002 (reference) 2003–2005 2006–2008 2009–2011 2012–2014 2015–2018	- 0.73 0.66 0.58 0.60 0.51	0.008 0.003 <0.001 <0.001 <0.001	(0.57, 0.92) (0.50, 0.87) (0.44, 0.76) (0.45, 0.79) (0.38, 0.67)
**Histology**			
Epithelioid (reference) Undefined Biphasic Sarcomatoid	- 1.11 1.49 2.85	0.149 0.032 <0.001	(0.96, 1.28) (1.03, 2.13) (1.99, 4.08)
**Receipt of Surgery**	0.84	0.024	(0.72, 0.98)
**Stage**			
Localized Regional Distant	0.88 1.01 1.10	0.359 0.925 0.336	(0.68, 1.15) (0.79, 1.30) (0.91, 1.33)

^a^ Age is categorized in 15-year intervals. HR: Hazard Ratio; CI: Confidence Interval.

## Data Availability

The data that support these findings are housed with the Surveillance, Epidemiology and End Results Program (SEER) 18 registry dataset from 2000-18.14 SEER is a program of the National Cancer Institute.
